# Physical activity, mental health, and healthy development in adolescents: mechanisms and implications for intervention

**DOI:** 10.3389/fpsyg.2026.1969485

**Published:** 2026-10-15

**Authors:** Liang Rui, Junming Zhang, Sa Li

**Affiliations:** 1 Henan Institute of Science and Technology, Xinxiang, China; 2 Mahasarakham University, Maha Sarakham, Thailand; 3 Qilu University of Technology, Jinan, China

**Keywords:** 24-h movement behaviors, adolescence, anxiety, depression, mental health, physical activity, school-based intervention, self-determination theory

## Abstract

Adolescence combines heightened neurobehavioral plasticity with a steep rise in the incidence of depressive and anxiety disorders. Habitual physical activity, by contrast, begins to fall much earlier, from about age seven, so adolescents enter the years of peak diagnostic risk already substantially less active than they were as children. That offset, rather than a coincidence of two curves, is the premise of this review. Meta-analytic syntheses report moderate improvements in depressive (SMD = −0.45, 95% CI −0.59 to −0.31) and anxiety symptoms (SMD = −0.39, 95% CI −0.61 to −0.17) after exercise interventions in samples aged 5 to 18 years, yet several large pragmatic trials have detected no benefit. This mini review synthesizes recent evidence on these effects, the mechanisms proposed to explain them, and why the evidence base remains internally inconsistent. We argue that the contradiction is largely resolvable: efficacy estimates come from a heavily overlapping pool of trials and are inflated by unblinded designs, self-reported outcomes, and passive comparators, whereas universal school-based trials recruit predominantly asymptomatic students and deliver far less activity than prescribed. Mechanisms span three nested levels, neurobiological, psychosocial and behavioral, but most mediation evidence is concurrent rather than temporally ordered. Read through self-determination theory and acceptance-based accounts of distress tolerance, tolerable difficulty appears to be part of how participation confers benefit, with implications for injury, dropout and elite youth sport. Priorities include adequately powered trials with active controls and process evaluation, compositional analysis of movement behaviors, and temporally ordered mechanistic designs.

## 1 Introduction

Adolescence is the window in which most lifetime mental disorders first emerge: peak age at onset across diagnoses is 14.5 years, with roughly half of first onsets before age 18 (Solmi et al., 2022). Physical activity follows a different timetable. Gateshead Millennium Cohort accelerometer data show activity declining from age 7 in every trajectory, with no evidence the decline begins at adolescence or exceeds childhood decline (Farooq et al., 2018); a systematic review places onset near age 7 and the steepest fall near age 9 (Habyarimana et al., 2025); and a Swedish birth cohort records a fall from 4.2 to 2.5 h per day between ages 5 and 11 (Lundgren et al., 2025). By 2016, 81.0% of students aged 11–17 years were insufficiently active (Guthold et al., 2020; Bull et al., 2020).

The two trajectories are offset by years rather than coincident: adolescents reach peak diagnostic risk having lost much of the activity they will lose. Declining activity may contribute causally to later risk; both curves may share upstream causes such as school structure, screen availability and sleep restriction; or early disengagement may itself mark vulnerability. Cohorts support a contributory role: activity at age 11 predicts lower incidence of psychiatric conditions by age 18 (HR = 0.88, 95% CI 0.79 to 0.98), with organized sport more protective than volume alone (Lundgren et al., 2025), and childhood activity trajectories predict young-adult health (Howie et al., 2020). This relocates the causal question to the years before adolescence, implying prevention trials may be sited too late.

Terminology follows established usage: physical activity is any skeletal-muscle movement that expends energy; exercise is its planned, structured and repetitive subset undertaken to improve fitness; and sport is rule-organized, usually group-based exercise (Caspersen et al., 1985). The distinction matters because trial evidence concerns exercise, epidemiological evidence concerns total activity, and some mechanisms below are specific to sport.

The question is not new: an early review of reviews found modest associations, cautioning that most evidence was correlational (Biddle and Asare, 2011), and a later systematic review proposed the still-dominant model grouping mechanisms as neurobiological, psychosocial and behavioral (Lubans et al., 2016). Researchers have since asked whether activity can prevent and treat adolescent mental health problems (Yan et al., 2024; Kang et al., 2026).

Fifteen years on, evidence remains inconsistent between rather than within study designs. Umbrella reviews of randomized trials report moderate symptom reduction for supervised exercise versus inactive controls (Singh et al., 2026), and cohorts report lower psychiatric incidence among more active children (Lundgren et al., 2025; Howie et al., 2020), whereas large pragmatic trials adding activity to school timetables report null effects (Wassenaar et al., 2021). We read this as informative rather than unresolved: the association is conditional on modality, dose, delivery quality, participant characteristics and social context.

This review therefore summarizes what recent syntheses do and do not establish, reassesses the evidence for each mechanistic level, extends it with accounts of motivation and acceptance that bear on adherence, dropout and challenge, and identifies the designs needed to specify for whom and under what implementation conditions activity helps.

## 2 Literature search and evidence appraisal

This narrative mini-review used a purposive, non-exhaustive search of PubMed, Web of Science, Scopus and PsycINFO (January 2011 to July 2026), combining terms for physical activity, exercise and sport with terms for depression, anxiety, wellbeing and mental health in young people. Priority went to systematic reviews, meta-analyses, umbrella reviews, adequately powered trials and prospective cohorts in English; full strategy and eligibility criteria appear in Supplementary material. No data were newly extracted or pooled; each estimate derives from the cited synthesis, whose limitations are inherited here.

Existing syntheses are numerous, recent, and largely contemporaneous, drawing on overlapping primary trials. Singh et al. (2026) cover 21 reviews and 375 RCTs; Purgato et al. (2024) recomputed 12 meta-analyses from 112 studies drawn from 18 existing ones. Agreement thus carries less evidential weight than independent replication, so we avoid calling it convergence, since bias propagates to every synthesis containing affected trials.

## 3 Evidence for mental health effects

### 3.1 Meta-analytic estimates and their shared evidence base

An umbrella review of 21 systematic reviews (375 RCTs; 38,117 participants aged 5–18 years) pooled 180 RCTs (*n* = 34,490) for depressive symptoms (SMD = −0.45, 95% CI −0.59 to −0.31, *I*^2^ = 71.4%) and 55 RCTs (*n* = 24,797) for anxiety (SMD = −0.39, 95% CI −0.61 to −0.17, *I*^2^ = 68.1%) (Singh et al., 2026). Following Cohen’s conventions (0.2, 0.5 and 0.8 as small, moderate and large), we describe magnitude separately from statistical significance. Two qualifications apply: the samples span childhood, not just adolescence, and high heterogeneity means each pooled value is a central tendency rather than an expected effect.

A second umbrella review recomputed 12 meta-analyses from 112 studies (*n* = 21,232), reaching the same directional conclusion but judging the association weak, with GRADE credibility moderate to very low (Purgato et al., 2024). Benefits are also reported for perceived stress, self-esteem and social competence (Fu et al., 2025), and in overweight or obese adolescents (Chen et al., 2024; Men et al., 2026). Given trial overlap, this partly shared evidence supports a modest average benefit of moderate certainty at best.

### 3.2 Null findings in pragmatic trials, and what they indicate

The Fit to Study cluster RCT added 10 min of high-intensity interval training per physical education lesson for one academic year in 18,261 English students aged 12–13, and found no improvement in fitness, cognition or mental health, with low adherence and high attrition (Wassenaar et al., 2021). A systematic review of school-based interventions likewise found no significant effects on depression, anxiety or distress, health-related quality of life excepted (Ditzen-Janotta et al., 2026).

Three non-exclusive explanations reconcile these findings with the meta-analytic literature. First, efficacy estimates are likely inflated: blinding is impossible, outcomes are mostly self-reported, comparators are usually waitlist or no treatment, and small trials dominate, favoring expectancy and small-study bias. Second, universal school trials recruit largely asymptomatic students, leaving little symptom variance to reduce. Third, implementation dilutes dose, and unreported process evaluation obscures whether interventions fail or simply go undelivered (Wassenaar et al., 2021; Ditzen-Janotta et al., 2026). Heterogeneous exposure measurement compounds all three. These explanations are testable: pooled effects should shrink as trials adopt active controls.

### 3.3 Modality and dose: signals that outrun their evidence

Open- and closed-skill activities show different cognitive profiles (Zhang et al., 2026), and closed-skill exercise produced more durable gains in subthreshold depression (Gu et al., 2024). A network meta-analysis of 58 trials (*n* = 4,887) ranked resistance exercise first for treating (SMD = −1.30, 95% CI −1.96 to −0.64) and preventing (SMD = −1.18, 95% CI −1.65 to −0.71) depression, and found anxiolytic effects in youth with (SMD = −0.98, 95% CI −1.50 to −0.45) and without (SMD = −0.47, 95% CI −0.66 to −0.29) depression (Zhang et al., 2023a). Another ranked resistance training first for depression and combined aerobic–resistance training first for anxiety, though its pairwise analysis found no significant anxiety effect (MD = −0.12, 95% CI −0.32 to 0.09) (Zhou S. et al., 2025). Yoga shows large effects in attention-deficit/hyperactivity disorder (ADHD) (Shenning et al., 2026), and three to four weekly 30–60 min sessions beyond 6 weeks are most consistently linked to improvement (Wang et al., 2022; Zhang et al., 2023a).

Such rankings warrant caution. An SMD of −1.30 exceeds plausible bounds for a behavioral intervention on symptoms, rests on few small trials, and carries a wide interval. Network estimates depend on indirect comparisons across differing studies, questioning transitivity and rank stability: the anxiety ranking arose absent any demonstrated pairwise effect, showing how readily rank order emerges from unsupportive data. Intensity is operationalized inconsistently, and duration confounds with adherence. Head-to-head trials varying a single dose parameter, not further indirect comparison, are needed.

### 3.4 Populations at elevated risk

In youth with overweight or obesity, higher moderate-to-vigorous activity at age 7 predicts fewer internalizing problems at ages 11 and 14 (Li et al., 2025), and lifestyle interventions improve depressive symptoms (SMD = −0.24) and quality of life (SMD = 0.24) (Zhou J. et al., 2025). In ADHD, umbrella-level evidence indicates improvements in inattention and inhibitory control (Dastamooz et al., 2023); a three-week program reduced stress and normalized salivary cortisol, but the effect had gone by three-month follow-up (Dastamooz et al., 2026), so maintenance is the harder problem. In autism spectrum disorder, activity reduces internalizing problems via sport friendship quality and social–emotional competence (Zhu et al., 2025), with benefits comparable across neurodevelopmental diagnoses (Liu et al., 2024a; Liu et al., 2024b).

These subgroup patterns admit more than one interpretation and are not evidence of causality. Larger effects where baseline symptoms, adiposity or self-regulatory difficulty are greatest resemble a ceiling-limited causal effect, but the pattern is equally consistent with regression to the mean in samples selected on elevated baseline scores, floor effects in asymptomatic participants, and differing scale properties across the range. They are separable in principle, since regression to the mean predicts comparable drift in an untreated elevated-symptom control arm whereas a ceiling-limited effect does not, but few trials report that comparison. The defensible conclusion is weaker: sample composition plausibly contributes to the universal-selective discrepancy without explaining away the null results (Table 1).

**Table 1:** Evidence patterns for physical activity and adolescent mental health.

Evidence stream or population	Main findings summarized in the review	Interpretation and principal limitations	Representative sources
Umbrella and meta-analytic syntheses	An umbrella review of 21 systematic reviews (375 RCTs; *n* = 38,117; ages 5–18) pooled 180 RCTs (*n* = 34,490) for depression (SMD = −0.45, 95% CI −0.59 to −0.31, *I*^2^ = 71.4%) and 55 RCTs (*n* = 24,797) for anxiety (SMD = −0.39, 95% CI −0.61 to −0.17, *I*^2^ = 68.1%). Benefits have also been reported for perceived stress, self-esteem and social competence.	The direction of effect is consistent, but certainty is graded weak to very low and heterogeneity is high. Unblinded participation, self-reported outcomes, passive comparators and small trials may inflate pooled estimates. The syntheses share many constituent trials, so agreement between them is not independent replication, and samples span ages 5–18 rather than adolescence alone.	Singh et al. (2026), Purgato et al. (2024), Fu et al. (2025)
Universal school-based interventions	The one-year Fit to Study cluster RCT (*n* = 18,261) found no improvement in fitness, cognition, or mental health. A systematic review of school-based programs found no significant effects on depression, anxiety, or psychological distress, with HRQoL as the main exception.	Predominantly asymptomatic samples provide limited symptom variance. Low adherence, high attrition, and a delivered dose below the prescribed dose make intervention failure difficult to distinguish from failure to intervene.	Wassenaar et al. (2021), Ditzen-Janotta et al. (2026)
Exercise modality and dose	Network meta-analyses ranked resistance exercise highest for treating (SMD = −1.30, 95% CI −1.96 to −0.64) and preventing (SMD = −1.18, 95% CI −1.65 to −0.71) depression, and combined aerobic–resistance training highest for anxiety. Programs of 3–4 sessions per week, 30–60 min per session, for more than 6 weeks were most consistently associated with improvement.	Rankings depend on indirect comparisons across small, heterogeneous studies; one anxiety ranking was produced despite a non-significant pairwise effect (MD = −0.12, 95% CI −0.32 to 0.09). Intensity is defined inconsistently and duration effects are confounded by adherence. Direct head-to-head dose-comparison trials are needed.	Zhang et al. (2023a), Zhou S. et al. (2025), Wang et al. (2022), Singh et al. (2026)
Adolescents with overweight or obesity	Higher moderate-to-vigorous physical activity at age 7 predicted fewer internalizing problems at ages 11 and 14. Multicomponent lifestyle interventions improved depressive symptoms (SMD = −0.24) and quality of life (SMD = 0.24).	Greater baseline risk may increase observable benefit, but regression to the mean, floor effects in low-symptom samples and scale properties are equally consistent with the pattern. Because many programs are multicomponent, the independent contribution of physical activity cannot always be isolated.	Li et al. (2025), Zhou J. et al. (2025), Chen et al. (2024), Men et al. (2026)
ADHD and other neurodevelopmental disorders	Exercise improves inattention, inhibitory control, and cognitive flexibility in ADHD. A 3-week intervention reduced perceived stress and normalized salivary cortisol, but the effect was absent at 3 months. Across neurodevelopmental disorders, pooled benefits were broadly comparable across diagnoses.	Maintenance is a more difficult problem than initial response. Integrated programming is plausible, but durability, diagnosis-specific mechanisms, and the influence of social context remain uncertain.	Dastamooz et al. (2023, 2026), Liu et al. (2024a, 2024b), Shenning et al. (2026)

ADHD, attention-deficit/hyperactivity disorder; HRQoL, health-related quality of life; RCT, randomized controlled trial; SMD, standardized mean difference.

## 4 Mechanisms

Following Lubans et al. (2016), candidate mechanisms, neurobiological, psychosocial and behavioral, are better treated as nested than competing: behavioral context determines how much activity is accumulated and displaced, psychosocial processes shape how it is experienced and sustained, and neurobiological processes provide the substrate through which it alters affect and cognition. The evidential imbalance the model’s authors identified, abundant work on self-perceptions and sparse work on the other two levels, has narrowed since 2016 but not closed. Table 2 summarizes these pathways, their limitations and implications for intervention.

**Table 2:** Multilevel mechanisms linking physical activity to adolescent mental health.

Level or pathway	Candidate processes	Current evidence and major caveat	Implications for intervention and research
Neurobiological	Network connectivity and efficiency; frontal activation; BDNF, kynurenine metabolism, growth hormone, TNF-α, HPA-axis regulation, and inflammatory signaling.	Changes in connectivity, electrophysiology, and peripheral biomarkers accompany symptom or cognitive improvement. Evidence remains indirect because samples are small, peripheral markers are not central measures, and mediators are often concurrent with outcomes (Heinze et al., 2021; Yan et al., 2026).	Use repeated multimodal measures and test whether mediator change precedes symptom change.
Psychosocial: individual	Self-esteem, self-concept, self-efficacy, resilience, attitudes, perceived control, and intention.	Self-perceptions and efficacy-related constructs are the best-supported psychosocial mediators, but shared self-report methods and weak temporal ordering may inflate indirect effects (Nguyen Ho et al., 2023; Shen et al., 2022; Zhuang et al., 2026).	Build mastery, competence, and autonomy support into programs; test mediation with temporally ordered measures.
Psychosocial: relational and contextual	Friendship quality, social–emotional competence, belonging, purpose, structure, and the climate created by coaches, peers, and parents.	Relational variables mediate internalizing problems, and team sport predicts fewer later symptoms. Evidence is largely qualitative, observational, or concurrent, and climate effects are rarely isolated (Zhu et al., 2025; Ooms et al., 2025; Graupensperger et al., 2021).	Prioritize inclusion, supportive coaching, and belonging rather than treating activity dose as the sole active ingredient.
Behavioral: 24-h movement composition	Physical activity, sleep, sedentary and screen time, timing, and behavioral substitution across the day.	Meeting activity, sleep, and screen-time recommendations is associated with lower distress, but joint adherence is rare. Time use is zero-sum, most evidence is cross-sectional, and reverse causation remains plausible (Sampasa-Kanyinga et al., 2021; Zhang et al., 2023b; Hossian et al., 2025).	Use compositional or isotemporal-substitution models and integrate activity with sleep and screen-time management.
Theoretical integration	Self-determination theory: competence, autonomy, relatedness. Social cognitive theory: reciprocal determinism. Achievement goal theory: mastery versus performance climate. Planned behavior: intention and perceived control. Salutogenesis: comprehensibility, manageability, meaningfulness.	Self-determination theory has the largest evidence base in physical education and youth sport and maps onto the psychosocial mediators identified above; planned behavior predicts uptake but not maintenance. The frameworks are rarely tested together (Ryan and Deci, 2000; Vasconcellos et al., 2020; Bandura, 1998; Nicholls, 1984; Shen et al., 2022; Super et al., 2021).	Deliver dose, motivation and meaning together; build autonomy support and mastery climate into delivery; assess adherence and participation quality, not dose alone.
Risk pathway: double-edged exposure	Sports injury, athletic-identity disruption, fear of reinjury, high training load, and early specialization.	Mental health and injury are bidirectionally associated, and strong athletic identity predicts worse depressive symptoms after injury, challenging a linear “more is better” model (Chow et al., 2026; Park et al., 2023; Hoang et al., 2025).	Embed safeguarding, load management, injury prevention, and post-injury psychological support within activity promotion.
Challenge and tolerable difficulty	Graded difficulty, survivable failure, mastery, distress tolerance, values-based commitment, and dropout.	Some but nonzero lifetime adversity predicts better later mental health and lower reactivity to new adversity, an inverted-U pattern (Seery et al., 2010). Evidence is from general developmental samples rather than sport, and the inverted-U has not been tested for sport-based challenge; most children leave organized sport before adolescence (Crane and Temple, 2015; Hayes et al., 2006).	Grade difficulty rather than minimize it; test whether challenge relates to benefit non-monotonically; target distress tolerance and valued action alongside enjoyment.

BDNF, brain-derived neurotrophic factor; HPA, hypothalamic–pituitary–adrenal; TNF-α, tumor necrosis factor-α.

### 4.1 Neurobiological mechanisms

Longer-term interventions alter frontal activation and electrophysiological indices alongside improvements in mental health and cognition (Heinze et al., 2021). In adolescents with subthreshold depression, 12 months of aerobic exercise reduced resting-state electroencephalographic connectivity while increasing global network efficiency, with two mediating pathways showing opposing associations with symptom change (Yan et al., 2026), suggesting an effect on network integration and segregation rather than any single region. Exercise also modulates kynurenine metabolism, growth hormone, tumor necrosis factor-α and brain-derived neurotrophic factor (Vancampfort et al., 2025; Millan-Domingo et al., 2026), buffers stress reactivity via hypothalamic–pituitary–adrenal axis regulation (Dastamooz et al., 2026), and may reduce pro-inflammatory signaling (Hu et al., 2023).

Peripheral biomarkers indirectly proxy central processes, mechanistic substudies are small, and mediators are measured concurrently with outcomes, so findings suggest plausible substrates rather than causal pathways.

### 4.2 Psychosocial mechanisms

Self-esteem, self-concept and self-efficacy are the best-supported psychosocial mediators linking activity to psychiatric symptoms (Nguyen Ho et al., 2023), and resilience has repeatedly emerged as a mediator (Zhuang et al., 2026). Sport friendship quality and social–emotional competence serially mediate the association with internalizing problems (Zhu et al., 2025), while qualitative synthesis identifies distraction, purpose, growth, and belonging as experiential mechanisms shaped by coaches, teammates, and parents (Ooms et al., 2025). Team sport prospectively predicts fewer symptoms (Graupensperger et al., 2021), and organized sport, unlike activity volume alone, was protective in a birth cohort (Lundgren et al., 2025).

Because mediators and outcomes are usually self-reported on the same occasion, shared method variance probably inflates estimated indirect effects. More consequentially, if benefit depends on competence and belonging, an identical dose delivered in an evaluative or exclusionary climate should yield little benefit, which is a testable account of why compulsory physical education so often fails. Climate is a manipulable ingredient, not a nuisance variable.

### 4.3 Behavioral mechanisms: activity within the 24-h movement composition

Meeting all three Canadian 24-Hour Movement Guidelines, at least 60 min of moderate-to-vigorous activity, no more than two hours of recreational screen time, and age-appropriate sleep (9–11 h at ages 5–13, 8–10 h at ages 14–17), is associated with lower psychological distress (Tremblay et al., 2016; Sampasa-Kanyinga et al., 2021) and prospectively predicts fewer anxiety and depressive symptoms 6 months later (Zhang et al., 2023b), yet joint adherence is rare (0–53.6% across studies; below 10% in 87% of them) (Hossian et al., 2025). We use the Canadian guidelines because they specify these behaviors as one integrated composition; World Health Organization recommendations are equivalent for activity (Bull et al., 2020).

Timing matters, though not straightforwardly. In Chinese adolescents, moderate-to-vigorous activity predicted better mental health 6 months later on both weekdays and weekends, more strongly on weekends, while light activity predicted higher weekend but lower weekday life satisfaction (Yan et al., 2024). This likely reflects displaced activity and constrained autonomy rather than harm, since weekday light activity rises with age as moderate-to-vigorous activity falls, suggesting incidental rather than sustained movement.

Time use is compositional and zero-sum, so activity effects reflect substitution for sleep or sedentary time. Treating these as independent predictors may misattribute benefit; compositional or isotemporal-substitution approaches are required. Because evidence is mostly cross-sectional and plausibly bidirectional, reverse causation remains a live alternative.

### 4.4 Theoretical integration

Levels of mechanism alone cannot explain uptake, maintenance or dropout, so we read the evidence through frameworks chosen for the question each answers, stating our grounds. Self-determination theory is the primary organizing account, its constructs aligning with psychosocial evidence and having the largest empirical base; a meta-analysis showing autonomy-supportive climates predict autonomous motivation, engagement and wellbeing (Ryan and Deci, 2000; Vasconcellos et al., 2020). Competence, autonomy and relatedness map onto section 4.2’s constructs, predicting continuation.

Three further frameworks are complementary rather than competing. Social cognitive theory supplies the reciprocal determination of self-efficacy, behavior and environment, explaining why individually targeted programs fail in unchanged settings (Bandura, 1998). Achievement goal theory distinguishes mastery from performance climates, the most specific account of how an identical activity can build or undermine perceived competence (Nicholls, 1984). The theory of planned behavior (Shen et al., 2022) is retained only for the proximal prediction of uptake, being weak on maintenance. The salutogenic model addresses meaning rather than motivation, proposing that sport strengthens sense of coherence (Super et al., 2021). Together these clarify the central problem: interventions must deliver dose, motivation and meaning at once.

### 4.5 Tolerable difficulty and the shape of the dose–response curve

A needs-based account carries an easily missed implication: removing difficulty is not the goal. In a national longitudinal sample, some but nonzero lifetime adversity predicted lower distress and higher life satisfaction than either high adversity or none, along with lower reactivity to later adversity, a quadratic pattern read as resilience acquired through manageable challenge (Seery et al., 2010). Sport supplies such challenge unusually well, offering one reading of three otherwise separate observations: team sport predicts later symptoms more reliably than activity volume; injury harms partly by withdrawing mastery while imposing uncontrollable difficulty; and most children leave organized sport before adolescence (Crane and Temple, 2015), where difficulty often stops being graded and becomes selective. Acceptance-based approaches treat willingness to experience discomfort in service of valued activity as the trainable target (Hayes et al., 2006). Both predict an inverted-U rather than monotonic relation between challenge and benefit, so programs minimizing difficulty to maximize participation may retain adolescents without benefiting them, a testable and untested claim.

## 5 Physical activity as a double-edged exposure

Participation also carries psychological risk: injury predicts poorer mental health, and poorer mental health predicts higher injury risk (OR = 1.54), a bidirectional relationship (Chow et al., 2026). Athletic identity becomes a vulnerability after musculoskeletal injury, stronger identification predicting more severe depressive symptoms (Park et al., 2023), with identity loss and reinjury fear implicated in anxiety, depression, and post-traumatic stress symptoms (Hoang et al., 2025). Among elite youth athletes, these conditions are common and patterned by gender, athletic identity, and relationships with coaches, parents and peers (Gwyther et al., 2024).

This complicates the dose–response picture at its upper end (section 4.5). The training volume and early specialization that optimize performance are not those that optimize mental health, and elite youth sport’s difficulty is typically neither graded nor freely chosen, with injury rendering it uncontrollable. Injury prevention, load management and post-injury psychological support therefore belong within, not beside, activity-based mental health promotion (Bergeron et al., 2024; Nielsen et al., 2025).

## 6 Discussion

### 6.1 Methodological priorities and the move toward precision

The evidence base remains dominated by cross-sectional, short-term designs: 87% of 24-h movement studies are cross-sectional, and only 2% are interventions (Hossian et al., 2025). Progress requires adequately powered, preregistered trials with active controls, blinded outcomes, intention-to-treat analysis, and fidelity evaluation (Wassenaar et al., 2021; Ditzen-Janotta et al., 2026). Follow-up beyond 6 months is rarely reported yet essential, as effects dissipate (Axelsdóttir et al., 2021; Dastamooz et al., 2026); syntheses should also quantify trial overlap.

Because effects are larger in boys and secondary-school students (Fu et al., 2025), and sex, age, weight status, and baseline severity moderate response (Singh et al., 2026; Zhou J. et al., 2025), stratified designs matched to preference and competence could individualize dosing. Wearables enable monitoring (Kang et al., 2026), though digital delivery requires evaluation.

### 6.2 Mechanistic and contextual expansion

Mechanistic research should combine neuroimaging, electrophysiology, molecular markers, and behavioral measures in longitudinal designs establishing that mediator change precedes symptom change (Belcher et al., 2021; Heinze et al., 2021; Yan et al., 2026). Mechanisms are likely age-dependent (Xue et al., 2025); since activity decline begins in childhood, prevention trials should increasingly target primary-school years. Evidence remains concentrated in high-income countries (Hossian et al., 2025), and durable change requires coordination across school, family and community levels (Bergeron et al., 2024; Nielsen et al., 2025).

### 6.3 Implications for practice

Pending better trials, activity should be neither promoted indiscriminately nor withheld. Schools may reasonably prioritize enjoyment, perceived competence and inclusion over intensity, since these determine whether any dose is sustained; clinicians working with symptomatic adolescents have the strongest basis for recommending structured, preference-matched exercise alongside monitoring of sleep and screen time; and sport organizations should treat injury prevention and post-injury support as integral to the wellbeing case for participation.

## 7 Conclusion

Physical activity is a credible, low-cost and broadly acceptable component of adolescent mental health promotion, supported by meta-analytic evidence of small-to-moderate benefit for depressive and anxiety symptoms, albeit evidence drawn from a partly shared body of trials, and by plausible mechanisms at three levels. On current evidence, it is not reliably effective as a universal intervention added to school schedules. Benefit appears to depend on a sufficient dose, in a modality and climate that sustain competence, belonging, and tolerable challenge, delivered to young people with room to improve, and on the fact that the decline that sets the stage begins years before the disorders it aims to prevent. Testing that proposition, rather than re-estimating an average effect, should be the field’s next objective.
