# Supplementary material for: Physical activity, mental health, and healthy development in adolescents: mechanisms and implications for intervention
Source: Front Psychol. 2026 Oct 15;17:1969485. doi: 10.3389/fpsyg.2026.1969485 (PMC13716951; doi:10.3389/fpsyg.2026.1969485)
Supplement: Supplementary file 1 [file Supplementary_file_1.DOCX]

**Supplementary Material**

**S1. Search strategy**

This mini review is argument-driven and narrative. The literature search was purposive: it was designed to identify the recent syntheses, adequately powered trials, and prospective cohorts on which the argument depends, not to enumerate the primary literature exhaustively. It was not conducted to systematic-review standards and is not reported as such.

Databases: PubMed, Web of Science Core Collection, Scopus and PsycINFO.

Window: 1 January 2011 to 31 July 2026. The start point is the year of the last major review of reviews in this field (Biddle and Asare, 2011), which the present review takes as its point of departure. The closing date excludes work published after 31 July 2026.

Language: English-language records only. This is a limitation; relevant work in other languages, including Chinese-language school-based intervention research, was not retrieved.

Concepts combined with AND, terms within each concept combined with OR:

• Exposure: “physical activity”, exercise, sport, “physical education”, “moderate-to-vigorous”, “resistance training”, “aerobic exercise”, “24-hour movement”.

• Outcome: “mental health”, depression, depressive, anxiety, wellbeing, “well-being”, “psychological distress”, “self-esteem”, resilience, internalizing.

• Population: adolescent, adolescence, youth, child, children, “school-aged”, teenager.

• Design filters applied at screening rather than in the query: “systematic review”, “meta-analysis”, “umbrella review”, “randomized controlled trial”, “cohort study”.

Reference lists of all retrieved syntheses were screened for further eligible records. Foundational theoretical and definitional sources necessarily predate the window and were retrieved by targeted lookup rather than by the dated search; these are Caspersen et al. (1985), Nicholls (1984), Bandura (1998), Ryan and Deci (2000), Hayes et al. (2006) and Seery et al. (2010).

**S2. Eligibility criteria**

Included: systematic reviews, meta-analyses, network meta-analyses and umbrella reviews of physical activity or exercise with mental health, psychosocial or wellbeing outcomes in participants aged approximately 5 to 18 years; randomized controlled trials of adequate size, in particular pragmatic and cluster-randomized trials delivered in schools; prospective cohort studies with repeated measurement of activity and later mental health outcomes; consensus statements from recognized bodies on youth sport and safeguarding; and mechanistic studies reporting neurobiological, psychosocial or behavioral mediators.

Excluded: single cross-sectional studies, except where cited to illustrate a methodological point; adult-only samples; studies of physical activity with purely physical outcomes; conference abstracts; and records without an English-language full text.

**S3. Evidence appraisal, and the overlap between syntheses**

No data were newly extracted, recomputed, or pooled. Every quantitative estimate in the manuscript is reported as given in the cited synthesis, together with its confidence interval where the source provides one, and the limitations of those syntheses are inherited rather than corrected. Effect-size magnitude is described using Cohen's conventions (standardized mean differences of approximately 0.2, 0.5 and 0.8 as small, moderate and large) and is reported separately from statistical significance.

The syntheses relied on are numerous, recent and largely contemporaneous, and therefore share a large proportion of their constituent primary trials. Singh et al. (2026) cover 21 systematic reviews and 375 randomized controlled trials; Purgato et al. (2024) recomputed 12 meta-analyses from 112 studies drawn from 18 existing meta-analyses; the network meta-analyses of Zhang et al. (2023a) and Zhou et al. (2025b) include many of the same trials again. None of these papers reports a quantified overlap with the others, so the degree of duplication cannot be stated numerically here. Two consequences are carried into the body of the review: agreement among these syntheses is not independent replication and is not described as convergence, and any bias operating in the shared constituent trials propagates to every synthesis that contains them.
